# Maximal standard uptake values of ^18^F-fluoro-2-deoxy-D-glucose positron emission tomography compared with Epstein-Barr virus DNA as prognostic indicators in de novo metastatic nasopharyngeal carcinoma patients

**DOI:** 10.1186/s12885-019-6106-2

**Published:** 2019-09-11

**Authors:** Xue-Song Sun, Yu-Jing Liang, Sai-Lan Liu, Qiu-Yan Chen, Shan-Shan Guo, Yue-Feng Wen, Li-Ting Liu, Hao-Jun Xie, Qing-Nan Tang, Xiao-Yun Li, Jin-Jie Yan, Lin-Quan Tang, Hai-Qiang Mai

**Affiliations:** 1 State Key Laboratory of Oncology in South China; Collaborative Innovation Center for Cancer Medicine, Sun Yat-sen University Cancer Center, 651 Dongfeng Road East, Guangzhou, 510060 People’s Republic of China; 2 Department of Nasopharyngeal Carcinoma, Sun Yat-sen University Cancer Center, 651 Dongfeng Road East, Guangzhou, 510060 People’s Republic of China

**Keywords:** Nasopharyngeal carcinoma, EBV DNA, SUVmax, Survival

## Abstract

**Background:**

This study aimed to evaluate the prognostic value of maximal standard uptake values (SUVmax) of ^18^F-fluoro-2-deoxy-D-glucose positron emission tomography (PET) comparing with Epstein-Barr virus (EBV) DNA levels in de novo metastatic nasopharyngeal carcinoma (NPC) patients.

**Methods:**

From December 2006 to December 2016, 253 de novo metastatic NPC patients assessed by PET/ computed tomography were involved in current study. SUVmax-T, SUVmax-N, and SUVmax-M referred to the SUVmax at the primary tumor, cervical lymph nodes, and metastatic lesions respectively. Overall survival (OS) was the primary endpoint.

**Result:**

Patients who died during the follow-up had significantly higher SUVmax-N, SUVmax-M, and EBV DNA level than those in the patients who were alive. SUVmax-N and SUVmax-M were positively correlated with EBV DNA level. The cut-off values of SUVmax-T, SUVmax-N, SUVmax-M, and EBV DNA were 17.0, 12.7, and 6.9, and 13,800 copies/mL respectively, which were determined by receiver operating characteristic (ROC) curve analysis. Patients with elevated SUVmax-N, SUVmax-M, and EBV DNA levels had a lower 3-year OS rate. In multivariate analysis, the independent prognostic factors of OS included EBV DNA, metastatic site, and locoregional radiotherapy application, while SUVmax was not an independent prognostic factor.

**Conclusion:**

In de novo metastatic NPC patients, higher SUVmax-N and SUVmax-M were associated with worse prognosis. However, the predictive ability of SUVmax-N and SUVmax-M was poorer than that of EBV DNA.

**Electronic supplementary material:**

The online version of this article (10.1186/s12885-019-6106-2) contains supplementary material, which is available to authorized users.

## Background

Nasopharyngeal carcinoma (NPC) is a unique malignancy, which has distinguishing features from other head and neck cancer in terms of epidemiology, geographic areas, population, and prognosis. In 2012, approximately 86,700 new cases of NPC were reported, accounting for 0.6% of all cancers and causing 50,800 deaths [1, 2]. The area with the highest incidence is southern China, especially provinces such as Guangdong, Hainan, Guangxi, Hunan, and Fujian [3]. NPC is sensitive to both radiation and chemotherapy. Therefore, radiotherapy is the fundamental treatment modality for NPC while cisplatin-based concurrent chemoradiotherapy is the standard treatment for locoregionally advanced NPC [4]. Owing to great improvements in both diagnosis and radiation techniques, the prognosis of patients with non-metastatic NPC is quite satisfactory nowadays and the overall survival exceeds 80% [5].

It has been estimated that 15% of NPC patients develop distant metastasis at the time of the first diagnosis, which is defined as de novo metastatic NPC [6]. The prognosis of de novo metastatic NPC patients is poor even when treated with the first-line palliative chemotherapy regimen [7]. However, the survival period varies greatly among these patients with different illness condition [8]. Therefore, it is necessary to apply effective biomarkers for early survival prediction to guide individualized interventions.

In locoregionally advanced NPC, plasma Epstein-Barr virus (EBV) DNA levels have been proven to the most important biomarker, and it has been widely applied for condition monitoring and prognosis prediction [9, 10]. Furthermore, Li et al. have demonstrated the prognostic value of EBV DNA in metastatic NPC [11]. Therefore, EBV DNA could facilitate risk stratification. The National Comprehensive Cancer Network (NCCN) guidelines recommend ^18^F-fluoro-2-deoxy-D-glucose (FDG) positron emission tomography and computed tomography (PET/CT) for the detection of distant metastases in stage III-IV NPC patients. The maximal standard uptake value (SUVmax), used for a (semi) quantitative analysis in PET/CT, was indicated to have remarkable prognostic value in head and neck cancers [12]. In patients with non-metastatic NPC, previous studies have reported that the SUVmax represents a significant predictive factor of clinical outcomes [13–16]. However, to our knowledge, there is no related research on the predictive value of SUVmax in metastatic NPC.

Based on these facts, we conducted a retrospective study with a large sample size to investigate the utility of SUVmax in predicting survival outcomes in patients with de novo metastatic NPC in comparison with the prognostic value of EBV DNA, which would provide important information for personalized treatment.

## Methods

### Patient population

**Fig. 1:**
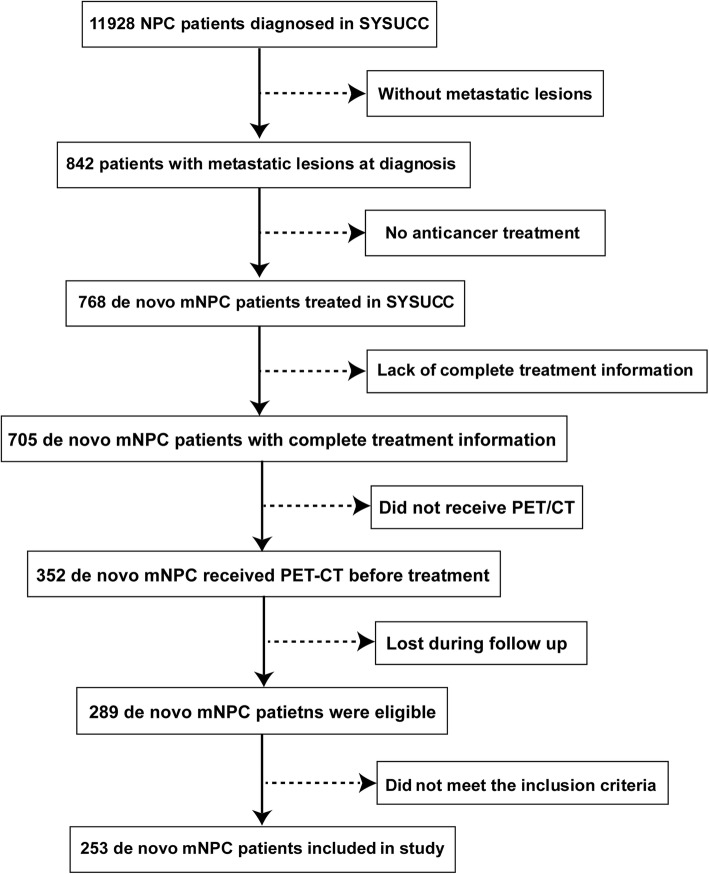
Flow chart showing patient inclusion

From December 2006 to December 2016, 253 patients with de novo metastatic NPC diagnosed at the Sun Yat Sen University Cancer Center (SYSUCC) were included in this study. The inclusion criteria were as follows: (1) histologically confirmed NPC; (2) evidence of distant metastasis assessed by PET/CT; (3) use of cisplatin-based palliative chemotherapy (PCT); (4) an initial Karnofsky performance score of > 70; (5) adequate renal and hepatic functions; and (6) no pregnancy, lactation, or second malignant disease. The flow chart is shown in Fig. 1. The analysis was approved by the clinical research ethics committee at SYSUCC.

### Diagnosis and treatment

Prior to diagnosis, the patients underwent a series of assessments including a physical examination, nasopharyngoscopy and biopsy, enhanced magnetic resonance imaging (MRI) of the nasopharynx and neck, and PET/CT. Enhanced MRI/computed tomography (CT) of the metastatic sites or other tests were considered by clinical oncologists when necessary. Palliative chemotherapy was administered for all patients. The common chemotherapy regimens were GP (gemcitabine [1000 mg/m^2^, d1,8] and cisplatin [20–30 mg/m^2^, d1–3]), TPF (docetaxel [60 mg/m^2^, d1] combined with cisplatin [60 mg/m^2^, d1] and 5-fluorouracil (500–800 mg/m^2^, d1–5]), PF (cisplatin [20–25/m^2^, d1–3] and 5-fluorouracil [800–1000 mg/m^2^, d1–5]), and TP (docetaxel [75 mg/m^2^, d1] plus cisplatin [20–30 mg/m^2^, d1–3]).

Chemotherapy was administered every 3 weeks intravenously. After PCT, 164 (64.8%) patients received locoregional radiotherapy (LRRT) using intensity-modulated radiation or two-dimensional conventional radiotherapy technique. The total dosage for radiation therapy was 68–70 Gy, five times a week from Monday to Friday, and 1.8–2.2 Gy each time.

### EBV DNA measurement and PET/CT imaging test

Before treatment, patients’ plasma EBV DNA was routinely measured by quantitative polymerase chain reaction as previously described [17]. PET/CT was performed based on procedural guidelines [18]. Forty-five to 60 min after the injection of FDG, PET/CT was performed from the head to the proximal thigh. Finally, with the use of CT data, the PET images were re-established [6]. SUVmax was defined as the highest activity concentration per injected dose per body weight with correction of radioactive decay. SUVmax-T, SUVmax-N, and SUVmax-M referred to the SUVmax at the primary tumor, cervical lymph nodes, and metastatic lesions respectively.

### Follow-up and outcome

After treatment, evaluations were conducted every 3 months for the first 3 years and then every 6 months thereafter until the patient died. Routine evaluations during follow-up included a physical examination; nasopharyngoscopy; enhanced MRI/CT of the nasopharynx, neck, and metastatic sites; chest X-ray/enhanced CT; and abdominal ultrasound/ enhanced CT. PET/CT or other tests were considered by clinical oncologists if necessary. The primary outcome of our study was overall survival (OS), and the definition of OS was the time from the date of diagnosis to the date of death from any cause.

### Statistical analysis

The Spearman correlation test was used to evaluate the correlation between SUVmax and EBV DNA levels, which were regarded as continuous variables. The values of SUVmax and EBV DNA between survivors and non-survivors were compared using the Mann–Whitney U-test. The cut-off points for continuous variables were chosen by receiver operating characteristic (ROC) curve analysis following the Metz method [19]. Patients’ baseline characteristics were evaluated using the Chi-square test. Survival probabilities between patients in different groups were evaluated using the Kaplan–Meier method with log-rank test. Cox proportional hazards regression model was applied in the step-wise multivariate analysis. All statistical analyses were performed using Statistical Package for Social Sciences 23.0 (IBM Corporation, Armonk, NY, USA). All statistical tests were two-tailed, and *p* < 0.05 was considered statistical significance.

## Results

### Patient characteristics

From December 2006 to December 2016, 253 de novo metastatic NPC patients were involved in the study. The median patient age was 47 years, and the male-to-female ratio was 5.8:1. In the cohort, 180 patients (71.1%) had one metastatic site, while 73 patients (28.9%) developed lesions at multiple metastatic sites on diagnosis. Other patient characteristics are listed in Table 1. The median follow-up period was 27.2 months [interquartile range (IQR) 15.9–39.9 months]. One hundred and thirty patients were dead at the last follow-up. The 3- and 5-year OS rates were 54.8 and 33.1%, respectively.

**Table 1:** Clinical characteristics

Characteristic	n(%)
Total	253
Gender	
Male	216(85.4%)
Female	37(14.6%)
Age (years)	
≤ 47	133(52.6%)
> 47	122(47.4%)
T stage #	
T1	12(4.7%)
T2	22(8.7%)
T3	133(52.6%)
T4	86(34.0%)
N stage #	
N0	4(1.6%)
N1	28(11.1%)
N2	94(37.2%)
N3	127(50.2%)
Metastatic sites	
Bone	116(45.8%)
Lung	28(11.1%)
Liver	14(5.5%)
Distant nodes	22(8.7%)
Multiple sites	73(28.9%)
LRRT use	
No	89(35.2%)
Yes	164(64.8%)

*Abbreviations*: *LRRT* locoregional radiotherapy

According to the 8th edition of the UICC/AJCC staging system

### Distribution of SUVmax and EBV DNA level in survivors and non-survivors

**Fig. 2:**
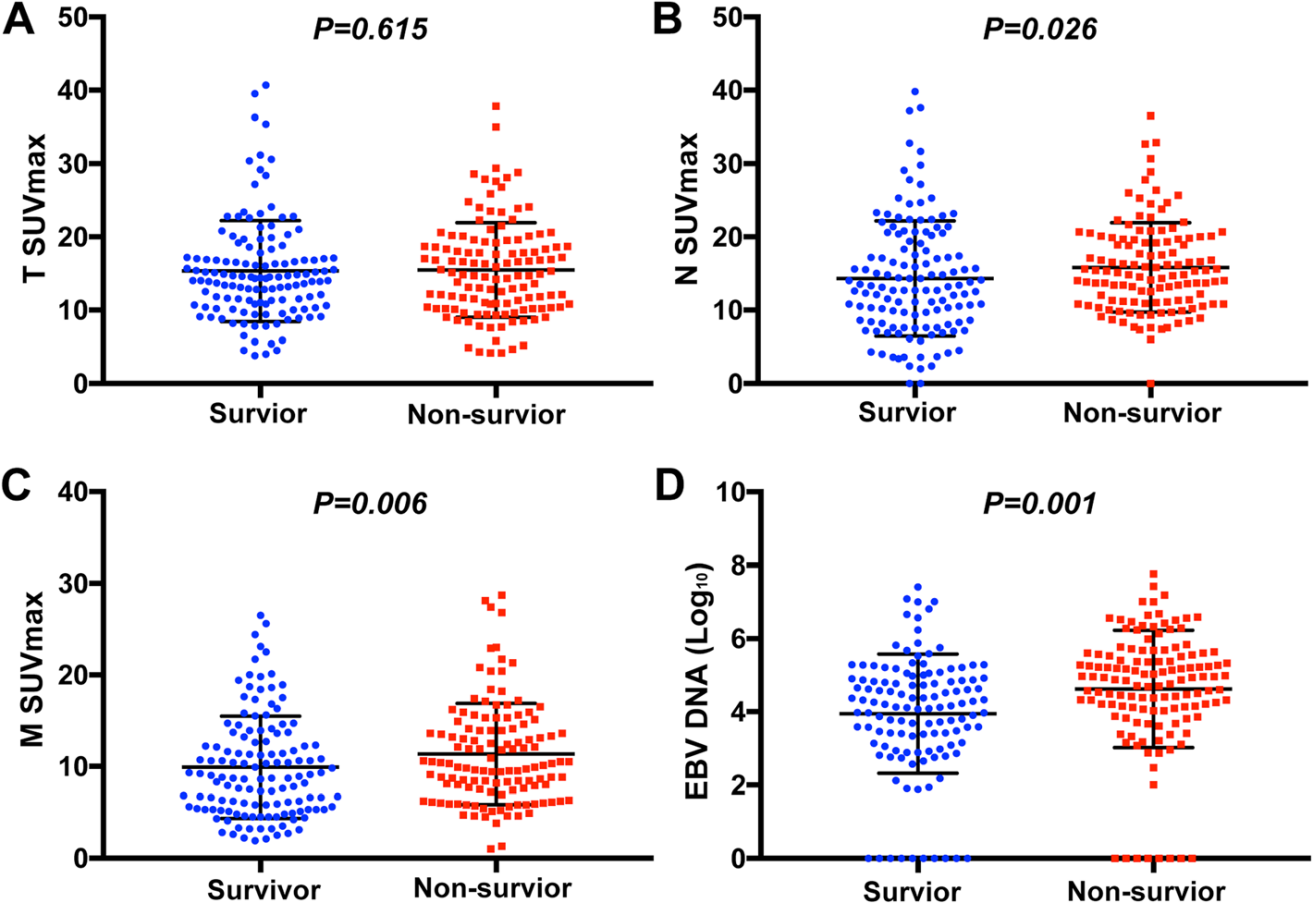
The distribution of different variables in the survivor and non- survivor groups. **a** SUVmax-T; **b** SUVmax-N; **c** SUVmax-M; and **d** EBV DNA level

As shown in Fig. 2, patients who died during the follow-up period had significantly higher SUVmax-N (*P* = 0.026) and SUVmax-M (*P* = 0.006) values than patients alive at the last follow-up. However, there was no obvious difference in SUVmax-T between survivors and non-survivors (*P* = 0.615). Because the copy number of EBV DNA is obviously asymmetric, we obtained the logarithm of EBV DNA with the base 10 to make the distribution of values more uniform on coordinate axes. Obviously, the median EBV DNA level was higher in patients who were dead compared to patients who were survival (*P* = 0.001).

### Correlation analysis between EBV DNA and SUVmax

**Fig. 3:**
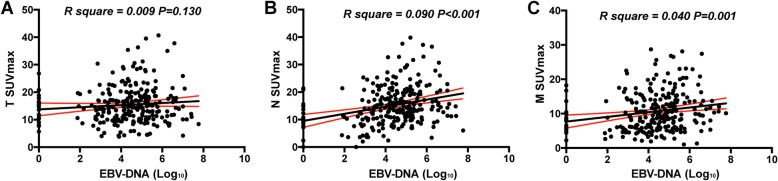
The correlations between SUVmax values and the EBV DNA level. **a** SUVmax-T; **b** SUVmax-N; and **c** SUVmax-M

A Spearman correlation analysis was performed between SUVmax and EBV DNA levels (log_10_). The SUVmax and EBV DNA levels were regarded as continuous variables. Interestingly, SUVmax-N (R square = 0.090, *P* < 0.001) and SUVmax-M (R square = 0.040, *P* = 0.001) were positively correlated with EBV DNA levels, while no significant correlation was found between SUVmax-T and EBV DNA levels (R square = 0.009, *P* = 0.130) (Fig. 3). It should be noted that although the results were significant, the correlations between SUVmax-N, SUVmax-M and EBV DNA levels were still weak.

### Cut-off value of EBV DNA and SUVmax

**Fig. 4:**
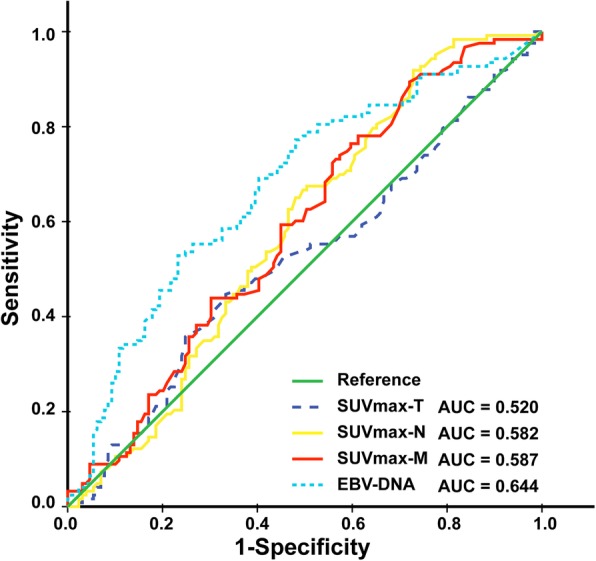
Receiver operating characteristic (ROC) curve analysis used to determine the cut-off SUVmax values and EBV DNA level

The ROC curve was applied to evaluate the ability of SUVmax and plasma EBV DNA to predict death and to choose the optimal cut-off value that showed the best trade-off between sensitivity and specificity in further analyses. According to the ROC analysis, the cut-off EBV DNA value was 13,800 copies/ml (sensitivity = 0.677, specificity = 0.528, area under curve [AUC] = 0.644) for OS (Fig. 4). The cut-off values of SUVmax-T, SUVmax-N, and SUVmax-M were 17.0, 12.7 and 6.9 respectively. The patient characteristics in different SUVmax and EBV DNA levels are shown in Table 2 and the follow-up durations of different subgroups are shown in Additional file 1: Table S1.

**Table 2:** Clinical characteristics grouped by different SUV and EBV DNA level

	SUVmax-T n(%)			SUVmax-N n(%)			SUVmax-M n(%)			EBV DNA n(%)		
Characteristic			*P* value			*P* value			*P* value			*P* value
Total	175	78		104	149		77	176		95	158	
Gender												
Male	148(84.6%)	68(87.2%)	0.701	88(84.6%)	128(85.9%)	0.857	67(87.0%)	149(84.7%)	0.702	78(82.1%)	138(87.3%)	0.274
Female	27(18.2%)	10(12.8%)		16(15.4%)	21(14.1%)		10(13.0%)	27(15.3%)		17(17.9%)	20(12.7%)	
Age (years)												
≤ 47	88(50.3%)	45(57.7%)	0.340	49(47.1%)	84(56.4%)	0.161	42(54.5%)	91(51.7%)	0.684	50(52.6%)	83(52.5%)	> 0.999
> 47	87(49.7%)	33(42.3%)		55(52.9%)	65(43.6%)		35(45.5%)	85(48.3%)		45(47.4%)	75(47.5%)	
T stage #												
T1	10(5.7%)	2(2.6%)	0.089	7(6.7%)	5(3.4%)	0.187	4(5.2%)	8(4.5%)	0.984	5(5.3%)	7(4.4%)	**0.008**
T2	19(10.9%)	3(3.8%)		7(6.7%)	15(10.1%)		7(9.1%)	15(8.5%)		15(15.8%)	7(4.4%)	
T3	93(53.1%)	40(51.3%)		49(47.1%)	84(56.4%)		39(50.6%)	94(53.4%)		50(52.6%)	83(52.5%)	
T4	53(30.3%)	33(42.3%)		41(39.4%)	45(30.2%)		27(35.1%)	59(33.5%)		25(26.3%)	61(38.6%)	
N stage #												
N0	4(2.3%)	0(0.0%)	0.525*	4(3.8%)	0(0.0%)	< 0.001*	2(2.6%)	2(1.1%)	0.332*	4(4.2%)	0(0.0%)	0.002*
N1	21(12.0%)	7(9.0%)		18(17.3%)	10(6.7%)		8(10.4%)	20(11.4%)		13(13.7%)	15(9.5%)	
N2	66(37.7%)	28(35.9%)		42(40.4%)	52(34.9%)		34(44.2%)	60(34.1%)		42(44.2%)	52(32.9%)	
N3	84(48.0%)	43(55.1%)		40(38.5%)	87(58.4%)		33(42.9%)	94(53.4%)		36(37.9%)	91(57.6%)	
Metastatic site sites												
Bone	87(49.7%)	29(37.2%)	0.056	58(55.8%)	58(38.9%)	**0.005**	31(40.3%)	85(48.3%)	**< 0.001**	47(49.5%)	69(43.7%)	**0.001**
Lung	20(11.4%)	8(10.3%)		13(12.5%)	15(10.1%)		13(16.9%)	15(8.5%)		17(17.9%)	11(7.0%)	
Liver	8(4.6%)	6(7.7%)		5(4.8%)	9(6.0%)		5(6.5%)	9(5.1%)		5(5.3%)	9(5.7%)	
Distant nodes	18(10.3%)	4(5.1%)		11(10.6%)	11(7.4%)		15(19.5%)	7(4.0%)		11(11.6%)	11(7.0%)	
Multiple sites	42(24.0%)	31(39.7%)		17(16.3%)	56(37.6%)		13(16.9%)	60(34.1%)		15(15.8%)	58(36.7%)	
LRRT use												
No	60(34.3%)	29(37.7%)	0.668	34(33.3%)	55(36.9%)	0.592	20(26.0%)	69(39.4%)	**0.045**	28(29.5%)	61(38.9%)	0.137
Yes	115(65.7%)	48(62.3%)		69(67.0%)	94(63.1%)		57(74.0%)	106(60.6%)		67(70.5%)	96(61.1%)	

*Abbreviations*: *LRRT* locoregional radiotherapy

According to the 8th edition of the UICC/AJCC staging system

The *P* value was calculated with the Pearson χ2 test or Fisher’s exact test (*)

Bold data referred to statistical significance (*P* < 0.05)

### Association between elevated SUVmax, EBV DNA levels and OS

**Fig. 5:**
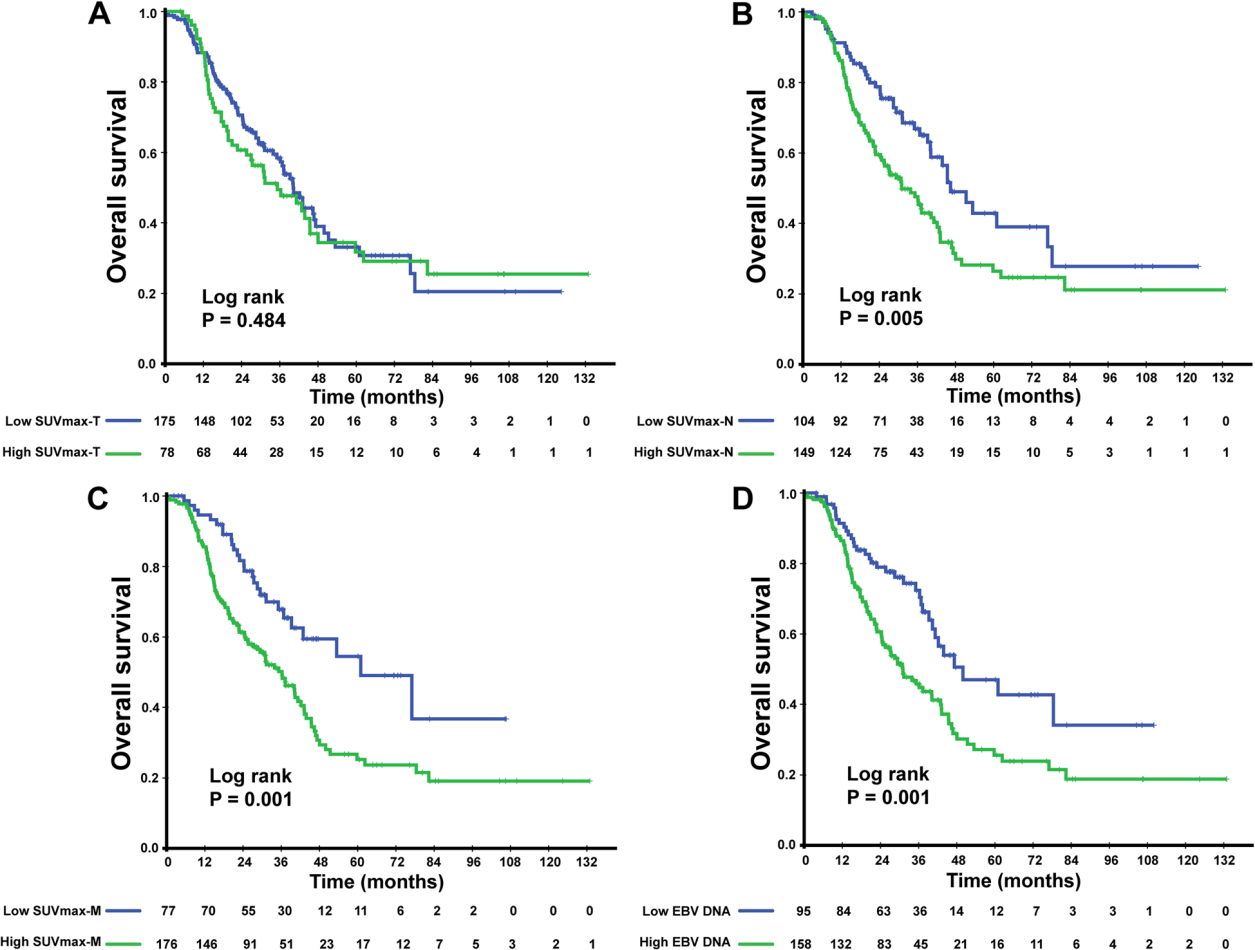
Kaplan–Meier survival curves comparing overall survival grouped by the cut-off SUVmax-T (**a**), SUVmax-N (**b**), and SUVmax-M (**c**) values and the EBV DNA level (**d**). *P* values were calculated using the log-rank test

We divided the patients into two different groups based on the cut-off SUVmax and EBV DNA values. In univariate analysis, patients with SUVmax-*N* > 12.7 showed a lower 3-year OS than patients with SUVmax-*N* ≤ 12.7 (65.1% vs. 46.5%, *P* = 0.005). Similarly, in comparison with patients with SUVmax-M > 6.9, patients with SUVmax-M ≤ 6.9 achieved a better survival condition (65.4% vs. 49.2%, *P* = 0.005). However, the 3-year OS was comparable among patients with different SUVmax-T levels (57.3% vs. 47.7%, *P* = 0.484). In terms of EBV DNA, the patients EBV DNA ≥ 13,800 copies/mL showed a worse survival condition than patients with lower EBV DNA level. The 3-year OS was 70.4 and 44.7%, respectively (*P* = 0.001). The Kaplan–Meier curve for OS is shown in Fig. 5.

### Multivariate analyses of prognostic factors

We further used three multivariate analysis models in our study (Table 3). In model 1, SUVmax-T, SUVmax-N, and SUVmax-M were involved in the analysis and only SUVmax-M was associated with OS (hazard ratio [HR]: 1.72, 95% confidence interval [CI]: 1.13–2.78, *P* = 0.012). In model 2, which was adjusted for EBV DNA level, both SUVmax-M and EBV DNA remained independent factors for OS. Finally, other risk factors (T stage, N stage, age, gender, metastatic site, and LRRT use) were considered. In model 3, EBV DNA level still remained an independent prognostic factor for OS (HR: 1.55, 95% CI: 1.03–1.23, *P* = 0.036) while SUVmax-M did not. Patients with multiple metastatic sites exhibited worse OS than patients with bone-only metastasis (HR, 1.87; 95% CI, 1.25–2.80, *P* = 0.002). Besides, LRRT use was a protective factor (HR, 0.51; 95% CI, 0.35–0.78; *P* < 0.001).

**Table 3:** Multivariate analyses

	Model 1		Model 2		Model 3	
Characteristic	HR (95%CI)	*P* value	HR (95%CI)	*P* value	HR (95%CI)	*P* value
SUVmax-T		NS		NS		NS
SUVmax-N	1.40(0.95–2.06)	0.087		NS		NS
SUVmax-M	1.72(1.13–2.78)	0.012	1.72(1.10–2.67)	0.017		NS
EBV-DNA			1.62(1.08–2.43)	0.020	1.55(1.03–2.32)	0.036
Metastatic site						
Lung vs. Bone					0.74(0.38–1.41)	0.357
Liver vs. Bone					1.10(0.52–2.32)	0.805
Distant nodes vs. Bone					0.50(0.18–1.39)	0.184
Multiple vs. Bone					1.87(1.25–2.80)	0.002
LRRT					0.51(0.35–0.78)	< 0.001

*Abbreviations*: *NS* non-significant, *HR* hazard ratio, *CI* confidence interval, *LRRT* locoregional radiotherapy

Backward step-wise multivariate analyses using Cox proportional hazard model was applied to select variables. Only variables that were significant associated with overall survival are presented

HRs were calculated for SUVmax-T (> 17.0 vs. ≤17.0); SUVmax-N (> 12.7 vs. ≤12.7); SUVmax-M (> 6.9 vs. ≤6.9); EBV DNA (> 13,800 copies/ml vs. ≤13,800 copies/ml); LRRT (Yes vs. No)

## Discussion

As far as we know, this is the first retrospective cohort study to explore the prognostic value of EBV DNA levels and SUVmax values in de novo metastatic NPC patients. Here, we found that SUVmax-N and SUVmax-M of ^18^F-FDG PET/CT had positive correlations with EBV DNA levels while SUVmax-T did not. Furthermore, SUVmax-N and SUVmax-M were related to the patients’ prognosis. EBV DNA level was superior to SUVmax in terms of its survival prediction value and remained an independent factor in multivariate analyses combining other risk factors.

EBV DNA level was an important biomarker for NPC as previous studies investigated [9, 10, 20]. Lin et al. demonstrated that higher EBV DNA levels (> 1500 copies/mL) prior to treatment or detectable levels after treatment were both related to lower OS for non-metastatic NPC patients [9]. The prognostic value is similar among metastatic and recurrent patients [11]. In our previous study, we established a prognostic nomogram combining EBV DNA level and other prognostic factors. The new model showed better discrimination than the traditional TNM stage [21]. Additionally, we demonstrated that the pretreatment plasma EBV DNA level was of great value in predicting distant metastasis for NPC patients, making the use of PET-CT more reasonable [6]. 18F-FDG uptake, which was measured by SUVmax, was related to the glucose metabolic rate of tumor cells. Previous studies have reported that non-metastatic NPC patients with lower SUVmax values achieved better survival rates [13, 16, 22]. Zhang et al. were the first group to develop an integrated prognostic model based on recursive partitioning analysis for DMFS, which incorporated SUVmax-N and N-classification [23]. In addition, Sher et al. demonstrated that NPC patients with higher SUVmax values had a worse 5-year OS, and the SUV75% on FDG PET could be used to identify patients benefiting from adjuvant chemotherapy [24].

In locoregionally advanced NPC, we have the verified the predictive value of SUVmax and EBV DNA level in previous study [25]. However, there was no relevant research on de novo metastatic NPC. In this study, we found that higher levels of SUVmax-N and SUVmax-M were significantly associated with a lower 3-year OS, but the SUVmax-T result was not. On the other hand, the EBV DNA level exhibited a superior predictive ability. Patients with a higher EBV DNA level suffered lower 3-year OS compared with other patients (70.4% vs. 44.7%, *P* < 0.001). Multifactorial Cox regression analysis suggested that for the OS, the independent prognostic factors included the EBV DNA level, metastatic sites, and LRRT use, but the SUVmax at any site was not involved. Additionally, we found that there were strong correlations between SUVmax-N, SUVmax-M values and the metastatic sites according to the Chi-square test (Table 2). According to the theory of multicollinearity, if there was a significant correlation between two prognostic factors in the Cox proportional hazards regression model, their predictive values would be influenced by each other. This may partially explain why these two factors did not remain independent prognostic factors in multivariate analysis. Our results showed that the EBV DNA level, which is the best biomarker so far, was more sensitive than SUVmax value for prediction of death in de novo metastatic NPC.

According to this study, a high level of SUVmax-N, SUVmax-M, and EBV DNA indicated worse prognosis in metastatic NPC patients at diagnosis. For these patients, closer follow-up examinations and early intervention were necessary. Cisplatin-based PCT has been established as the standard treatment regimen for metastatic NPC patients [7, 26–28]. Recently, several studies verified that LRRT could further improve patients’ survival when combined with PCT in metastatic NPC patients [8, 29]. Our results were consistent with these previous studies, which demonstrated that LRRT was a significant protective factor in multivariate analysis. Nevertheless, the current regular treatment therapy may not be adequate to improve the OS for high-risk patients. A new treatment method urgently needs to be identified. On the basis of this fact, our group initiated a worldwide, multicenter, phase III clinical study of cisplatin and gemcitabine with or without PD-1 antibody (toripalimab) in patients with recurrent or metastatic NPC (NCT 03581786). We are looking forward to the findings.

Several limitations existed in the current study. First, this was a retrospective study and the selection bias could not be eliminated. Second, all patients in current study were from one treatment center, and the pathology type of most patients was WHO type III. Third, the global standards for EBV DNA measurement are different, which need for further standardization. A prospective study from multiple institutions is needed to confirm our results.

## Conclusion

De novo metastatic NPC patients with high levels of SUVmax-N and SUVmax-M at diagnosis had a poor prognosis. Pre-EBV DNA level showed a stronger predictive ability than SUVmax and was the independent prognostic factor.

## Additional file

Additional file 1: **Table S1.** Follow-up durations of different subgroups (DOCX 51 kb)

## Data Availability

The datasets used and/or analyzed during the current study are available from the corresponding author on reasonable request.

## Abbreviations

AUC: Area under curve
CI: Confidence interval
CT: Computed tomography
EBV: Epstein–Barr virus
FDG: F-fluoro-2-deoxy-D-glucose
HR: Hazard ratio
KPS: Karnofsky performance score
LRRT: Locoregional radiotherapy
MRI: Magnetic resonance imaging
NCCN: National Comprehensive Cancer Network
NPC: Nasopharyngeal carcinoma
OS: Overall survival
PCT: Palliative chemotherapy
PET-CT: Positron emission tomography and computed tomography
ROC: Receiver operating characteristic
SUVmax: Maximal standard uptake value
SYSUCC: Sun Yat Sen University Cancer Center
