# Supplementary material for: Maximal standard uptake values of 18F-fluoro-2-deoxy-D-glucose positron emission tomography compared with Epstein-Barr virus DNA as prognostic indicators in de novo metastatic nasopharyngeal carcinoma patients
Source: BMC Cancer. 2019 Sep 11;19:908. doi: 10.1186/s12885-019-6106-2 (PMC6740035; doi:10.1186/s12885-019-6106-2)
Supplement: Supplementary file 1 — Table S1. Follow-up durations of different subgroups (DOCX 51 kb) [file 12885_2019_6106_MOESM1_ESM.docx]

Table S1: Follow-up durations of different subgroups

| Subgroup | Follow-up duration Mean (range) | P value |
| --- | --- | --- |
| **SUVmax-T** |  |  |
| ≤17.0 | 30.1 (1-124) months | 0.170 |
| >17.0 | 34.9 (3-132) months |  |
| **SUVmax-N** |  |  |
| ≤12.7 | 34.6 (3-124) months | 0.079 |
| >12.7 | 29.5 (1-132) months |  |
| **SUVmax-M** |  |  |
| ≤6.9 | 35.1 (3-106) months | 0.108 |
| >6.9 | 30.1 (1-132) months |  |
| **EBV DNA** |  |  |
| ≤13800 copies/ml | 34.1 (4-110) months | 0.176 |
| >13800 copies/ml | 30.1 (1-132) months |  |

The P value was calculated with the T test.
